# Intravenous thrombolysis in patients with acute ischaemic stroke and active cancer (the ITACA-stroke study)

**DOI:** 10.1007/s00415-025-13458-6

**Published:** 2025-10-23

**Authors:** Virginia Cancelloni, Giovanni Merlino, Gianluca Costamagna, Patrik Michel, Marialuisa Zedde, Ilaria Grisendi, Rosario Pascarella, Marina Padroni, Gianvito Barbella, Walter Ageno, Elisa Ruggeri, Simona Sacco, Alessio Troisi, Raffaele Ornello, Marina Mannino, Valeria Terruso, Roberto Sperotto, Arianna Cella, Mariarosaria Valente, Eugenia Rota, Manuel Cappellari, Francesco Valletta, Mara Zenorini, Matteo Foschi, Antonio Genovese, Tiziana Tassinari, Valentina Saia, Valeria Caso, Cecilia Becattini, Giorgio Maraziti, Ilaria Leone de Magistris, Michele Venti, Andrea Alberti, Alessandro Pezzini, Michelangelo Mancuso, Nicola Giannini, Marco Baldini, Elisa Grifoni, Luca Masotti, Michele Romoli, Sebastiano Giacomozzi, Georgios Tsivgoulis, Lina Palaiodimou, Aikaterini Theodorou, Odysseas Kargiotis, George Ntaios, Orestis Tsiotsikas, Efstathia Karagkiozi, Anastasia Adamou, Paraskevi Valarakou, Nemanja Popovic, Sanela Popovic, Pietro Caliandro, Aurelia Zauli, Giuseppe Reale, Marco Moci, Laetitia Yperzeele, Susanna Maria Zuurbier, Alessandro Padovani, Andrea Morotti, Piergiorgio Lochner, Maurizio Acampa, Rossana Tassi, Francesca Guideri, Alberto Chiti, Maurizio Paciaroni, Maria Giulia Mosconi

**Affiliations:** 1 Stroke Unit and Division of Cardiovascular Medicine, University of Perugia, Perugia, Italy; 2 SOSD Stroke Unit, Department of Head-Neck and Neuroscience, Azienda Sanitaria Universitaria Friuli Centrale (ASUFC), Udine, Italy; 3 Department of Medicine (DMED), University of Udine, Udine, Italy; 4 Stroke Centre, Neurology Service, Department of Clinical Neurosciences, Lausanne University Hospital and University of Lausanne, Lausanne, Switzerland; 5 Dino Ferrari Centre, Department of Pathophysiology and Transplantation (DEPT), University of Milan, Milan, Italy; 6 Neurology Unit, Stroke Unit, Azienda Unità Sanitaria Locale-IRCCS di Reggio Emilia, Reggio Emilia, Italy; 7 Neuroradiology Unit, Azienda Unità Sanitaria Locale-IRCCS di Reggio Emilia, Reggio Emilia, Italy; 8 Stroke Unit, Azienda Ospedaliero-Universitaria di Ferrara, Ferrara, Italy; 9 Department of Medicine and Surgery, University of Insubria, Varese, Italy; 10 Department of Biotechnological and Applied Clinical Sciences, University of L’Aquila, L’Aquila, Italy; 11 Department of Neurology and Stroke Unit, Azienda Ospedaliera Ospedali Riuniti Villa Sofia-V. Cervello, Palermo, Italy; 12 Neurology Unit, S. Giacomo Hospital, ASL AL, Novi Ligure, Italy; 13 Stroke Unit, Azienda Ospedaliera Universitaria Integrata, Verona, Italy; 14 Department of Neuroscience, Neurology Unit, S. Maria delle Croci Hospital, AUSL Romagna, Ravenna, Italy; 15 Stroke Care Program, Emergency Department, Parma University Hospital, Parma, Italy; 16 Neurology and Stroke Unit, Santa Corona Hospital, Pietra Ligure (SV), Italy; 17 Department of Medicine and Surgery, University of Parma, Parma, Italy; 18 Stroke Care Program, Department of Emergencies, Parma University Hospital, Parma, Italy; 19 Neurology Unit, Clinical and Experimental Medicine Department, Azienda Ospedaliero-Universitaria Pisana, Pisa, Italy; 20 Internal Medicine II and Stroke Unit, S. Giuseppe Hospital, Empoli, Italy; 21 Neurology and Stroke Unit, Bufalini Hospital, AUSL Romagna, Cesena, Italy; 22 Department of Biomedical and Neuromotor Sciences (DIBINEM), Alma Mater Studiorum-University of Bologna, Bologna, Italy; 23 Second Department of Neurology, School of Medicine, National and Kapodistrian University of Athens, “Attikon” University Hospital, Athens, Greece; 24 Stroke Unit, Metropolitan Hospital, Piraeus, Greece; 25 Department of Internal Medicine, Faculty of Medicine, School of Health Sciences, University of Thessaly, Larissa, Greece; 26 Clinic of Neurology, University Clinical Centre of Vojvodina, Faculty of Medicine, University of Novi Sad, Novi Sad, Serbia; 27 Stroke Unit, Neurology Unit, Fondazione Policlinico Universitario A. Gemelli IRCCS, Rome, Italy; 28 Unit of High Intensity Neurorehabilitation, Fondazione Policlinico Universitario A. Gemelli IRCCS, Rome, Italy; 29 Stroke Unit and Neurovascular Centre, Department of Neurology, Antwerp University Hospital, Antwerp, Belgium; 30 Translational Neurosciences Research Group, Faculty of Medicine and Health Sciences, University of Antwerp, Antwerp, Belgium; 31 Department of Continuity of Care and Frailty, Neurology Unit, ASST Spedali Civili di Brescia, Brescia, Italy; 32 Neurology Unit, Department of Clinical and Experimental Sciences, University of Brescia, Brescia, Italy; 33 Department of Neurology, Saarland University Medical Centre, Homburg, Germany; 34 Stroke Unit, Department of Medical Sciences, Surgery and Neurosciences, University of Siena, Siena, Italy; 35 Neurology Unit, Ospedale Apuano, Massa Carrara, Italy; 36 Unit of Clinical Neurology, Department of Neuroscience and Rehabilitation, University of Ferrara, Ferrara, Italy

**Keywords:** Acute ischaemic stroke, Thrombolysis, Cancer, Risk of bleeding, Functional outcome

## Abstract

**Background and aim:**

Current data regarding the effect of intravenous thrombolysis (IVT) in patients with acute ischaemic stroke (AIS) with concomitant malignancy remain unclear. This study aimed to evaluate the efficacy and safety of IVT in patients with AIS and concomitant active cancer.

**Methods:**

The ITACA-stroke study was a retrospective observational study based on prospective registries of a multicentre international collaboration. Patients with a diagnosis of active cancer and AIS, treated or not with IVT, were enrolled. Multivariable logistic regression analysis was performed to identify independent predictors for functional outcomes (mortality and good functional outcome) and haemorrhagic events at 90 days. A propensity score matching (PSM) analysis compared IVT and no-IVT patients.

**Results:**

A total of 521 patients were included, 225 were treated with IVT and 296 were not treated with IVT. IVT was directly correlated with good functional outcome (OR: 2.56, 95% CI 1.45–4.52, *p* = *0.001*), intracranial haemorrhage (ICH) (OR: 4.85, 95% CI: 2.09–11.22, *p* < *0.*001) any haemorrhagic event (OR: 4.13, 95% CI: 1.99–8.57, *p* < *0.001*), and inversely correlated with mortality (OR: 0.41, 95% CI: 0.22–0.74, *p* = *0.003*). In the PSM comparison, IVT was directly associated with good functional outcome (OR: 2.09, 95% CI: 1.16–3.77, *p* = *0.014*) and inversely correlated with mortality (OR: 0.31, 95% CI: 0.15–0.64, *p* = *0.001*), but was not directly associated with haemorrhagic outcomes.

**Conclusions:**

Treatment with IVT could be beneficial in patients with AIS and active cancer in terms of functional outcome and mortality. An association between IVT and the risk of haemorrhagic events was less clearly defined in this sample.

**Supplementary Information:**

The online version contains supplementary material available at 10.1007/s00415-025-13458-6.

## Introduction

Cancer and acute ischaemic stroke (AIS) represent leading causes of death, morbidity and long-term disability worldwide. It is estimated that up to 10% of hospitalised ischaemic stroke patients have comorbid cancer. Additionally, in the 2 years after AIS, another 3–5% of patients receive a new cancer diagnosis [1]. Apart from traditional ischaemic stroke risk factors and mechanisms, different cancer-related risk factors exist: prothrombotic state and coagulopathy, endothelial dysfunction, non-bacterial endocarditis, direct tumour compression, meningeal dissemination, treatment-related vascular toxicity and procedural complications [2–4].

Intravenous thrombolysis (IVT) currently represents the only approved systemic reperfusion treatment for AIS. However, the AIS treatment in patients with concomitant active cancer poses unique challenges, including the need to balance the risk of recurrent stroke and other thromboembolic events with that of haemorrhagic complications associated with revascularisation therapy.

Current international guidelines on revascularisation therapies for AIS [5] suggest that cancer should not be an absolute contraindication for thrombolytic treatment. This recommendation is based on observational studies, corresponding to a very low quality of evidence. Recent data from systematic reviews and meta-analyses of observational studies regarding the treatment of ischaemic stroke patients with and without concomitant active cancer suggested that in patients with cancer, IVT may not be associated with an increase in intracerebral and major systemic bleedings. Moreover, no significant differences between cancer and non-cancer patients in terms of modified Rankin Scale (mRS), favourable outcome and mortality were found [6–8].

Therefore, the safety and efficacy of revascularisation therapy, and in particular IVT in ischaemic stroke patients with concomitant active cancer, remain unclear. Moreover, no studies have directly compared the impact of IVT versus no-IVT on functional outcomes and bleeding events in patients with comorbid cancer. Our study aimed to evaluate the efficacy (in terms of functional outcome and mortality) and safety (in terms of bleeding complications) of IVT in patients with AIS and concomitant active cancer, directly comparing treated and not-treated patients.

## Materials and methods

### Study design and population

The ITACA study (intravenous thrombolysis in patients with acute ischaemic stroke and active cancer) was a retrospective observational study of a multicentre international collaboration involving 28 stroke centres across Europe. Each centre provided retrospective data regarding patients based on inclusion and exclusion criteria derived from their local prospectively consecutive registries. Data were collected from September 2023 to January 2024. Patients with a diagnosis of active cancer and AIS, treated or not with IVT, were enrolled. Active cancer was defined as follows: (1) a diagnosis of cancer that occurred within 6 months before acute ischaemic stroke or during hospitalisation for the index event, (2) treatment for cancer with surgery, chemotherapy or radiotherapy or a combination of them within 6 months before acute ischaemic stroke, and (3) patients with a previously known cancer with a diagnosis of recurrence or metastasis during the 6 months before acute ischaemic stroke. Patients with acute ischaemic stroke without a concurrent diagnosis of active cancer were excluded.

### Data collection

The following baseline variables were collected: age, sex, information on ongoing antithrombotic therapy (with both anticoagulants and antiplatelets) and statin treatment on admission. The following cerebrovascular risk factors were also collected: history of previous stroke or transient ischaemic attack (TIA), known or newly diagnosed atrial fibrillation, history of arterial hypertension (blood pressure of ≥ 140/90 mmHg at least twice before stroke or treatment with antihypertensive drugs), diabetes mellitus (fasting glucose level ≥ 126 mg/dL preprandial on two measurements, glucose level ≥ 200 mg/dL post-prandial, or HbA1c ≥ 6.5%, or diabetic treatment), hyperlipidaemia (total cholesterol ≥ 200 mg/dL or triglyceride ≥ 140 mg/dL or lipid-lowering therapy), current alcohol abuse (≥ 300 g per week), current cigarettes smoking, history of ischaemic heart disease (myocardial infarction, angina, existence of multiple lesions on thallium heart isotope scan, or evidence of coronary disease on coronary angiography), and history of cardiac heart failure.

On admission, the following laboratory data were collected: glycaemia, total and LDL cholesterol, blood count (comprehensive of haemoglobin, haematocrit, and platelet count), creatinine, blood coagulation structure (including partial thromboplastin time, PTT, prothrombin time, PT, and international normalised ratio, INR). According to local laboratory references, normal values were considered as follows: PT 9.8–14.3 s, PTT 25–35 s, INR 0.8–1.2.

Regarding the acute cerebrovascular event, information regarding National Institute of Health Stroke Scale (NIHSS) score on admission, aetiological classification of acute ischaemic stroke according to the TOAST classification system [17], and administration of intravenous thrombolysis were collected. IVT was defined as the administration of alteplase at the dosage of 0.9 mg/Kg that was delivered as per standard local protocol. On admission, either a brain computed tomography (CT) without contrast or a brain magnetic resonance imaging (MRI) scan was performed on all patients to exclude intracranial haemorrhage. A second brain CT scan or MRI was also performed 24–72 h after stroke onset on all patients.

As concerns the concomitant neoplastic disease, information regarding the site of active cancer, the presence of lymphonodal involvement and cancer staging according to TNM classification [9] was obtained. The site of active cancer was categorised as either haematological or solid, with specific sites further classified as colorectal, lung, breast, genitourinary, gynaecological, pancreatic/hepatobiliary, upper gastrointestinal, brain, bone/soft tissue, skin and other. Administration of any past or active treatments, such as surgery, radiation, pharmacological therapy (chemotherapy or immunotherapy) or a combination, was also investigated. Past treatments were defined as any treatment administered over six months before acute ischaemic stroke; current treatments were defined as any treatment administered within six months before acute ischaemic stroke.

### Outcome evaluation

Follow-up initiated at the time of the index event and lasted 3 months. Follow-up visits and outcome adjudication were performed by local investigators in a non-blinded fashion.

Primary outcome measures were mortality, disability and good functional outcome at 90 days. Disability was defined as a mRS score of 3–5. Good functional outcome was defined as a mRS of 0–2.

Secondary outcome measures were the occurrence of intracranial haemorrhage (ICH), symptomatic intracranial haemorrhage (sICH) and extracranial severe bleeding. ICH, that included intracerebral bleeding, subdural, or subarachnoid haemorrhage, was defined on CT as any degree of hyperdensity and on MRI as hypointensity on axial T1- and T2-weighted images. ICH was defined as symptomatic if accompanied by a decline in neurological status with a deterioration in NIHSS score of > or = 4 [10]. The definition of major extracranial bleeding as released by the International Society of Thrombosis and Haemostasis (ISTH) was a reduction in the haemoglobin level of 2 g per decilitre or more, the requirement of a blood transfusion of at least two units, or symptomatic bleeding in either a critical area or organ.

### Statistical analysis

We compared baseline characteristics of patients with active malignancy treated and not treated with IVT using the *χ*2 test for categorical variables and the Mann–Whitney U test for continuous variables. Patient characteristics are summarised for continuous variables as mean ± SD if they were normally distributed and as median and interquartile range (IQR) if they were not normally distributed. Categorical variables were presented as absolute numbers and percentages. Primary analysis was logistic regression analysis, which compared the risk of the study outcomes between treated and untreated cohorts. Results were reported as odds ratios (OR) and 95% confidence intervals (CIs). Multivariable analysis was performed using logistic regression to investigate any independent predictors of the primary and secondary outcomes. The independent variables included in the multivariable models were: age, male sex, previous stroke or TIA, presence of atrial fibrillation, history of hypertension, history of diabetes mellitus, hyperlipidemia, current alcohol use, current smoking habit, history of congestive heart failure (CHF), history of myocardial infarction (MI), antiplatelets on admission, oral anticoagulants on admission, statins on admission, NIHSS on admission, presence of metastasis and lymphonodal involvement as concerns active malignancy, and administration of IVT. Sensitivity analysis used logistic regression to investigate independent predictors for primary outcomes, considering cancer-related variables such as the presence of metastasis, linfonodal involvement and cancer sites.

A further analysis using propensity score matching (PSM) was performed to compare patients treated and not treated with IVT. This score was calculated by including selected variables from the univariate analysis, using backwards stepwise analysis with a 0.1 level as a screening criterion (covariate adjustment using the propensity score). Subsequently, matching was carried out through a nearest neighbour matching method with a 1:1 ratio across the groups and a calliper at 0.01 without replacement. The cluster-robust variance was used to estimate the standard errors. The imbalance between groups was checked before and after matching visually, estimating the standardised/normalised difference and the Chi-square test. Statistical significance was defined as *p* < 0.05. Data were analysed using R version 4.3.1.

## Results

### Characteristics of the study population

The final cohort comprised 521 patients with acute ischaemic stroke and active cancer. Of the 521 patients, 225 received IVT, and 296 did not. Regarding the study outcomes, 15 patients were lost during the 3-month follow-up (5 in the IVT group and 10 in the non-IVT group). Baseline characteristics of the study population are summarised in Table 1. Patients who received IVT had greater stroke severity on hospital admission (based on NIHSS score on admission) and a lower prevalence of metastasis.

**Table 1:** Characteristics of patients treated and not treated with intravenous thrombolysis

	IVT [n. 225]	No IVT [n. 296]	*p*
Age	74.1±11.6	72.9±11.1	0.3
Sex (M)	126 (56%)	160 (54.1%)	0.7
Previous stroke or TIA	36 (16%)	63 (21.3%)	0.1
Atrial fibrillation	71 (31.5%)	83 (28%)	0.4
Hypertension	151 (67.1%)	187 (63.2%)	0.4
Diabetes mellitus	50 (22.2%)	60 (20.3%)	0.6
Hyperlipidemia	84 (37.3%)	100 (33.8%)	0.4
Alcoholism	18 (8%)	29 (9.8%)	0.7
Current smoker	43 (19.1%)	49 (16.6%)	0.5
MI history	26 (11.5%)	48 (16.2%)	0.3
CHF history	20 (8.9%)	20 (6.8%)	0.4
NIHSS on admission	9.2±5.3	7.8±6.5	**0.007**
Antiplatelet on admission	73 (32.4%)	88 (29.7%)	0.5
Anticoagulant on admission	29 (12.9%)	55 (18.6%)	0.1
Statins on admission	62 (27.6%)	69 (23.3%)	0.3
Linfonodal involvement	124 (55.1%)	157 (53%)	0.9
Metastasis	87 (38.7%)	160 (54.1%)	**0.001**

The bold value in table indicate the statistically significant results (*p*<0.05)

In Table S1 in the Supplemental Material, study population characteristics as concerns the site of active cancer and TOAST classification of AIS are summarised. The most frequent active cancers were those involving lung (22.6%), genitourinary tract (18.4%), breast (13.2%), and colorectal tract (10.4%); other cancers involved thyroid, parotid gland, pharynx, larynx, and mouth. Information regarding the aetiology of acute ischaemic stroke based on the TOAST classification system was available for 512 patients: the most frequent aetiologies were undetermined aetiology (159/512 patients, 31.1%), cardioembolism (131/512 patients, 25.6%), and stroke of other determined aetiologies (84/512 patients, 16.4%).

### Rates and predictive factors of ischaemic and bleeding events

After a follow-up time of 3 months, 277 patients (56.4%) were deceased or disabled (mRS of 3–6), while 214 patients (43.6%) showed a good functional outcome (mRS of 0–2).

The multivariable analysis on the administration of IVT (Table S2 in the Supplemental Materials) showed that the clinical severity of acute ischaemic stroke (based on NIHSS score) was directly correlated with treatment with IVT (OR: 1.04, 95% CI 1.00–1.08 for each point increase). Previous MI history (OR: 0.46, 95% CI: 0.22–0.98), concomitant therapy with anticoagulants on admission (OR: 0.47, 95% CI: 0.22–1.00) and presence of metastatic active cancer (OR: 0.54, 95% CI: 0.330.89) were inversely correlated with the administration of IVT.

The univariate analysis (Table S3 in the Supplemental Materials) demonstrates that patients with good functional outcomes at 90 days showed less severe ischaemic strokes (based on NIHSS score), a lower prevalence of metastasis and concomitant use of anticoagulants on admission, and were more frequently treated with IVT. As concerns primary and secondary outcomes at 3 months, the univariate model demonstrated that IVT patients had a higher prevalence of good functional outcome and a lower prevalence of death or disability, as well as of any haemorrhagic events (ICH, sICH and extracranial bleeding). The univariate analysis regarding primary and secondary outcomes in IVT and no-IVT patients is reported in Table 2.

**Table 2:** Univariate analysis on primary and secondary outcomes at 3 months in patients treated and not treated with intravenous thrombolysis

	**IVT** **[n.220]**	**No IVT** **[n.286]**	**O.R. [95% CI]**	***p***
Mortality	44 (20%)	90 (31.5%)	0.54 [0.36–0.82]	**0.002**
Good functional outcome	114 (51.8%)	100 (35.0%)	2.00 [1.40–2.86]	**<0.001**
Death or disability	102 (46.4%)	175 (61.2%)	0.55 [0.38–0.78]	**<0.001**
ICH	34 (15.5%)	14 (4.9%)	3.55 [1.85–6.80]	**<0.001**
sICH	9 (4.1%)	3 (1.1%)	4.02 [1.08–15.04]	**0.04**
Extracranial severe bleeding	13 (5.9%)	6 (2.1%)	2.93 [1.10–7.84]	**0.03**

The bold value in table indicate the statistically significant results (*p*<0.05)

From the logistic regression analysis, the administration of IVT was found to be directly correlated with good functional outcome (OR: 2.56, 95% CI: 1.45–4.52, *p* = 0.001) and inversely correlated with mortality (OR: 0.41, 95% CI: 0.22–0.74, *p* = 0.003), at 90 days. NIHSS score on admission (OR: 0.81, 95% CI: 0.76–0.86) and concomitant use of anticoagulants (OR: 0.27, 95% CI: 0.10–0.69) were inversely correlated with good functional outcome at 90 days. Mortality was found to be directly correlated to NIHSS score on admission (OR: 1.09, 95% CI: 1.05–1.15) but inversely correlated to previous history of CHF (OR: 0.21, 95% CI: 0.06–0.74).

**Fig. 1:**
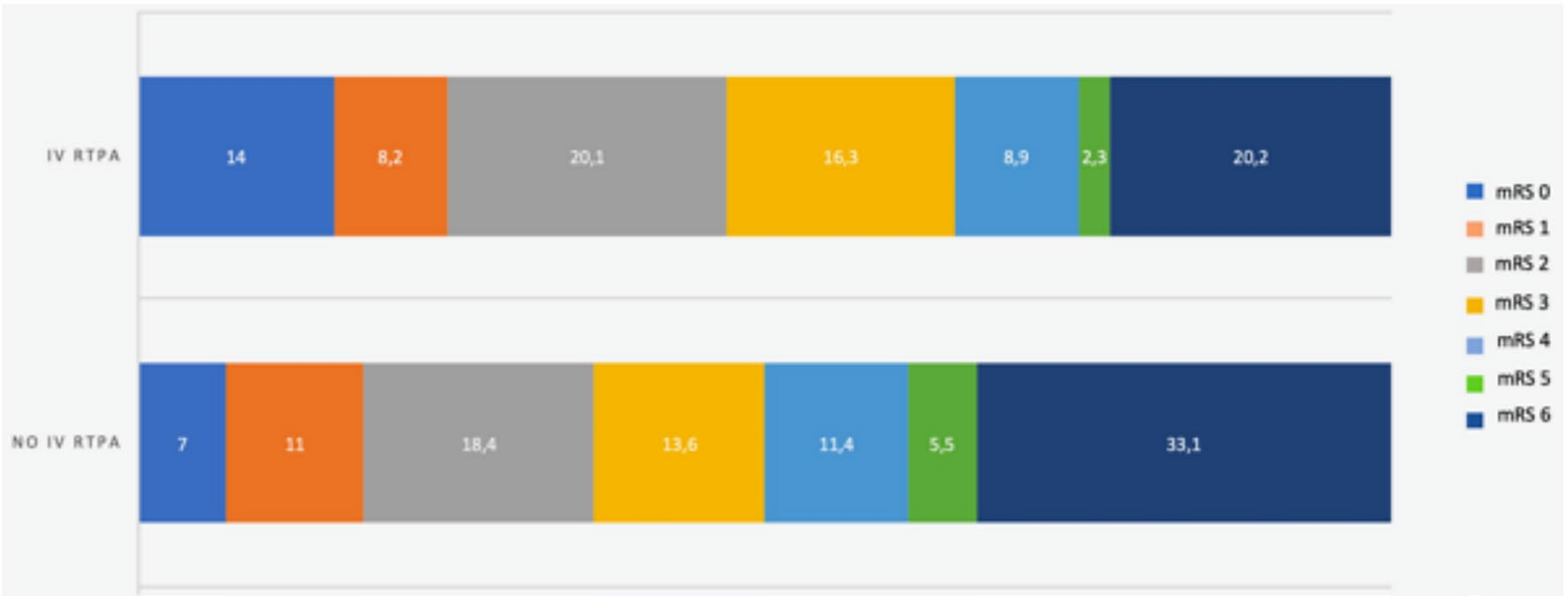
Modified Rankin scale (mRS) in patients treated and not treated with intravenous rt-PA. *IVT* intravenous thrombolysis, *TIA* transient ischaemic attack, *MI* myocardial infarction, *CHF* congestive heart failure, *NIHSS* National Institute of Health Stroke Scale, *ICH* intracranial haemorrhage, *sICH* symptomatic intracranial haemorrhage

Because of the small number of haemorrhagic outcome events, we performed multivariate analysis on any haemorrhagic event and ICH that occurred at 90 days. The administration of IVT was directly correlated with the risk of any haemorrhagic event (OR: 4.13, 95% CI: 1.99–8.57, *p* < 0.001) and ICH (OR: 4.85, 95% CI: 2.09–11.22, *p* < 0.001), at 90 days. Risk of haemorrhagic event at 90 days was significantly associated with female sex (OR:0.45, 95% CI: 0.23–0.89), history of previous stroke or TIA (OR:2.34, 95% CI: 1.06–5.18), NIHSS on admission (OR:1.07, 95% CI: 1.01–1.12), and anticoagulation therapy on admission (OR:3.42, 95% CI: 1.31–8.93). Treatment with anticoagulants on admission (OR: 4.51, 95% CI: 1.55–13.16) was found to be a positive predictive factor also for ICH. Results from the multivariate analysis regarding primary and secondary outcomes are reported in Table 3 in the present manuscript and Tables S4, S5, S6 and S7 in the Supplemental Materials. Frequencies of primary and secondary outcomes event based on different primary sites of active cancer, are reported in Table S8 in the Supplemental Materials. Figure 1 shows the distribution of mRS in patients treated and not treated with IVT.

**Table 3:** Multivariable analysis on association between intravenous thrombolysis and primary and secondary outcomes at 3 months

	OR [95% CI]	*p* value
Good functional outcome	2.56 [1.45–4.52]	**0.001**
Mortality	0.41 [0.22–0.74]	**0.003**
Any haemorrhagic event	4.13 [1.99–8.57]	**<0.001**
Intracranial haemorrhage	4.85 [2.09–11.22]	**<0.001**

Adjusted for: age, sex (M), previous stroke/transient ischaemic attack, atrial fibrillation, hypertension, diabetes mellitus, hyperlipidemia, alcoholism, current smoking, history of myocardial infarction, congestive heart failure, NIHSS on admission, antiplatelets on admission, anticoagulants on admission, statins on admission, linfonodal involvement and presence of metastasis.

The bold value in table indicate the statistically significant results (*p*<0.05)

Sensitivity analysis showed that the presence of metastasis was inversely correlated with good functional outcome (OR: 0.52, 95% CI: 0.31–0.88, *p* = 0.02) and directly correlated with mortality (OR:2.12, 95% CI: 1.21–3.73, *p* = 0.009). The presence of linfonodal involvement and the cancer site did not influence primary outcomes (Table 4).

**Table 4:** Sensitivity analysis on primary outcomes considering cancer-related variables

	Good functional outcome		Mortality	
	O.R. [95% CI]	*P*	O.R. [95% CI]	*p*
Linfonodal involvement	0.85 [0.49–1.50]	0.57	1.30 [0.70–2.41]	0.41
Metastasis	0.52 [0.31–0.88]	**0.02**	2.12 [1.21–3.73]	**0.009**
Cancer site				
Lung	0.56 [0.03–9.68]	0.69	0.60 [0.04–10.13]	0.73
Genitourinary	0.41 [0.02–7.27]	0.54	0.35 [0.02–6.10]	0.47
Breast	1.15 [0.06–20.86]	0.93	0.18 [0.01–3.25]	0.24
Colorectal	0.60 [0.03–11.05]	0.73	0.36 [0.02–6.47]	0.49
Pancreatic/hepatobiliary	0.30 [0.02–5.56]	0.42	1.19 [0.07–20.89]	0.91
Upper gastrointestinal	0.21 [0.01–3.93]	0.29	0.45 [0.02–8.20]	0.59
Hematological	0.49 [0.02–10.43]	0.64	0.25 [0.01–6.07]	0.39
Other sites	0.85 [0.05–16.21]	0.92	0.43 [0.02–7.99]	0.57

Adjusted for: age, sex (M), NIHSS on admission and IVT

The bold value in table indicate the statistically significant results (*p*<0.05)

After PSM, 91 IVT patients and 91 no-IVT patients were compared. In the Supplemental Materials, the distribution of propensity scores in IVT and no-IVT patients and the Love plot regarding the balance of the variables included in the PSM analysis (Figs. 1 and 2, respectively) are reported. The characteristics of the patients after PSM are reported in Table S9 in the Supplemental Materials.

Concerning functional outcomes, the multivariate analysis after PSM showed that the administration of IVT was directly correlated with good functional outcome (OR: 2.09, 95% CI: 1.16–3.77, *p* = 0.014) and inversely correlated with mortality at 90 days (OR: 0.31, 95% CI: 0.15–0.64, *p* = 0.001), but was not significantly associated with the incidence of any haemorrhagic event, (OR: 1.0, 95% CI: 0.42–2.37, *p* = 1.00), ICH (OR: 2.87, 95% CI: 0.92–8.87, *p* = 0.068), or extracranial major bleeding (OR: 1.65, 95% CI: 0.45–6.05, *p* = 0.45). Data concerning the multivariate analysis of primary and secondary outcomes after PSM are reported in Table 5.

**Table 5:** Multivariable analysis on primary and secondary outcomes at 3 months after propensity score matching

	IVT (n. 91)	No IVT (n. 91)	Total (n. 182)	OR (95% CI)	*p* value
Good functional outcome	47 (52.8%)	31 (34.8%)	78 (43.8%)	2.09 (1.16–3.77)	**0.014**
Mortality	14 (15.6%)	33 (37.1%)	47 (26.3%)	0.31 (0.15–0.64)	**0.001**
Any haemorrhagic event	11 (12.1%)	11 (12.1%)	22 (12.1%)	1.0 (0.42–2.37)	1.000
ICH	13 (14.3%)	5 (5.5%)	18 (9.9%)	2.87 (0.92–8.87)	0.068
Extracranial severe bleeding	5 (5.5%)	6 (3.4%)	11 (4.5%)	1.65 (0.45–6.05)	0.45

The bold value in table indicate the statistically significant results (*p*<0.05)

## Discussion

In this multicenter international cohort study, we found that the administration of IVT in patients with AIS and concomitant active cancer was directly correlated with good functional outcome and inversely correlated with mortality at 90 days. The same results were obtained after PSM, where 91 patients who had received IVT were compared to 91 untreated patients. The multivariable model showed that IVT was significantly associated with both ICH and any haemorrhagic event. However, this data was not confirmed in the logistic regression after PSM, where IVT did not represent a predictive factor for haemorrhagic outcomes.

Recently, a systematic review and meta-analysis including 11 observational studies [8] showed that administration of IVT was not associated with a significant increase in the incidence of disability (random effect OR: 0.72; 95% CI: 0.35–1.49) or mortality (random effect OR: 1.26; 95% CI: 0.91–1.75) and ICH (random effect OR: 1.35; 95% CI: 0.85–2.14) between cancer and non-active cancer stroke patients. Similar results were reported in two other recent systematic reviews and meta-analyses [6, 7]. Differently fromprevious studies, which compared the safety-efficacy profile of IVT in patients with and without active cancer, our study cohort comprised only patients with active cancer. The increased risk of haemorrhagic events in patients treated with IVT evidenced in the logistic regression, could be associated not only with the treatment itself but also with cancer-related coagulopathy, endothelial dysfunction and cancer treatment-related vascular toxicity, which often characterise this category of patients. [2]

In our cohort, lung (22.6%), genitourinary (18.4%), breast (13.2%) and colorectal (10.4%) represented the most frequent sites of active cancer. This is in line with previous studies, which reported that patients diagnosed with lung, urogenital, breast and gastrointestinal cancers showed a higher stroke incidence [15, 16].

Sensitivity analysis showed that cancer site was not associated with mortality or with good functional outcome. As expected, both primary outcomes were directly correlated with the presence of metastasis.

Current available data underline how different types of cancer show different bleeding risk profiles. Bleeding incidence is reported to be higher in patients affected by skin, gastrointestinal and genitourinary cancers, as well as in metastatic or advanced-stage diseases [11, 12]. Because of the large number of cancer sites collected and the few rates of bleeding events, we decided not to perform an adjunctive analysis on the association between cancer sites and bleeding events, to avoid creating a dispersion of the results. However, we reported the frequencies of both primary and secondary outcomes in the six most frequent cancer sites. Similarly to previous data, haemorrhagic events were more frequent in patients affected by upper gastrointestinal and genitourinary cancer.

In our cohort, higher NIHSS on admission and the absence of metastatic cancer influenced the administration of IVT as positive factors. Moreover, higher NIHSS on admission was also directly correlated to all haemorrhagic outcome events at 90 days. As reported in previous studies [13, 14], patients with active cancer show more severe strokes than patients without cancer, probably leading to an increased risk of bleeding and haemorrhagic transformation secondary to IVT. As evidenced in our study and reported in previous data [11], concomitant treatment with oral anticoagulants represents a positive predictive factor for any haemorrhagic outcome event and ICH.

As concerns stroke classification, the most frequent aetiologies of AIS according to the TOAST classification system [17] in our cohort were undetermined aetiology (31.1%) and cardioembolism (25.6%). Prevalence of AF in patients with cancer is reported to be up to 20% and may be promoted by the presence of cancer itself as well as by cancer treatments [18]. On the other hand, stroke in cancer patients can result from several mechanisms, such as coagulation disorders, infections, direct tumour effect and the effect of therapeutic and diagnostic interventions, making the exact determination of stroke aetiology challenging [4].

Our study has limitations. It was an observational, non-randomised study, and consequently, a selection bias of the studied populations could not be excluded, despite the PSM approach. Undoubtedly, patients not treated with IVT may have more aggressive cancer types or a higher grade of cancer. Even if information regarding the presence of metastasis was collected, these factors may have influenced the patient’s functional outcomes and created a selection bias in our results. As mentioned above, because of the few rates of bleeding events, analysis on the association between cancer site and secondary outcomes was not performed, so conclusions about the risk of haemorrhagic events depending on specific cancer location cannot be derived. Moreover, data regarding specific cancer subtypes, which are known to be associated with different bleeding risk profiles, were not collected. Finally, some variables included in the PSM analysis show an absolute standard deviation > 0.2, which should imply a suboptimal matching. However, with the method reported in the manuscript, we obtained the best balance for most of the variables except for 5 (see Love plot).

A strength of the study was the investigation of the IVT safety-efficacy profile only in patients affected by active malignancy, reducing the heterogeneity of the study cohort. Moreover, despite being a retrospective study, patients were collected prospectively and in a multicenter modality.

## Conclusions

Our results suggest that the administration of IVT could be beneficial in patients with AIS and concomitant active cancer in terms of functional outcome and survival. Multivariate analysis after PSM did not confirm a statistically significant association between IVT and haemorrhagic events. When deciding about acute revascularisation therapy in this particular fragile population, caution seems appropriate, and the evaluation of the risk–benefit ratio is mandatory for all patients.

## Supplementary Information

Below is the link to the electronic supplementary material.

Supplementary file1 (DOCX 181 KB)

## Data Availability

The datasets generated during and/or analysed during the current study are available from the corresponding author on reasonable request.
