# Supplementary material for: Intravenous thrombolysis in patients with acute ischaemic stroke and active cancer (the ITACA-stroke study)
Source: J Neurol. 2025 Oct 23;272(11):723. doi: 10.1007/s00415-025-13458-6 (PMC12549749; doi:10.1007/s00415-025-13458-6)
Supplement: Supplementary file 1 — Supplementary file1 (DOCX 181 KB) [file 415_2025_13458_MOESM1_ESM.docx]

|  | **n [tot. 521]** | **IVT [n. 225]** | **No IVT [n. 296]** |
| --- | --- | --- | --- |
| **Site of active cancer** | | | |
| Lung | 118 (22.6%) | 48 (21.3%) | 70 (23.6%) |
| Genitourinary | 96 (18.4%) | 46 (20.4%) | 49 (16.6%) |
| Breast | 69 (13.2%) | 35 (15.6%) | 34 (11.5%) |
| Colorectal | 54 (10.4%) | 25 (11.1%) | 29 (9.8%) |
| Pancreatic/hepatobiliary | 49 (9.4%) | 16 (7.1%) | 33 (11.1%) |
| Upper gastrointestinal | 42 (8.1%) | 15 (6.7%) | 27 (9.12%) |
| Haematological | 36 (6.9%) | 16 (7.1%) | 20 (6.8%) |
| Gynaecological | 24 (4.6%) | 7 (3.1%) | 17 (5.7%) |
| Skin | 13 (2.5%) | 6 (2.7%) | 7 (2.4%) |
| Brain | 4 (0.8%) | 3 (1.3%) | 1 (0.3%) |
| Bone/soft tissue | 1 (0.2%) | 0 (0.0%) | 1 (0.3%) |
| Other | 15 (2.9%) | 7 (3.1%) | 8 (2.7%) |
| **TOAST classification of acute ischaemic stroke** | | | |
| Cardioembolism | 131 (25.6%) | 59 (26.2%) | 72 (24.3%) |
| Large artery disease | 72 (14.1%) | 23 (10.2%) | 49 (16.6%) |
| Small vessel disease | 66 (12.9%) | 29 (12.9%) | 37 (12.5%) |
| Stroke of other determined aetiologies | 84 (16.4%) | 30 (13.3%) | 54 (18.2%) |
| Stroke of undetermined aetiology (negative/incomplete evaluation or multiple aetiologies) | 159 (31.1%) | 75 (33.3%) | 84 (28.4%) |

**Table S1.** Characteristics of patients at baseline. IVT intravenous thrombolysis.

|  | **O.R. [95% CI]** | ***p value*** |
| --- | --- | --- |
| Age | 1.01 [0.99 - 1.04] | 0.22 |
| Sex (M) | 0.90 [0.52 – 1.56] | 0.71 |
| Previous stroke/TIA | 0.76 [0.41 – 1.41] | 0.38 |
| Atrial fibrillation | 0.87 [0.40 – 1.87] | 0.72 |
| Hypertension | 1.00 [0.60 – 1.67] | 1.00 |
| Diabetes mellitus | 1.14 [0.65 – 2.00] | 0.66 |
| Hyperlipidemia | 1.03 [0.58 – 1.84] | 0.93 |
| Alcoholism | 0.90 [0.41 – 1.97] | 0.80 |
| Current smoker | 1.09 [0.60 – 1.97] | 0.78 |
| MI history | 0.46 [0.22 – 0.98] | **0.04** |
| CHF history | 1.22 [0.52 – 2.87] | 0.64 |
| NIHSS on admission | 1.04 [1.00 – 1.08] | **0.05** |
| Antiplatelets on admission | 1.34 [0.73 – 2.46] | 0.35 |
| Anticoagulants on admission | 0.47 [0.22 – 1.00] | **0.05** |
| Statins on admission | 1.12 [0.57 – 2.19] | 0.74 |
| Linfonodal involvement | 0.81 [0.48 – 1.35] | 0.41 |
| Metastasis | 0.54 [0.33 – 0.89] | **0.02** |

**Table S2.** Multivariate analysis on the administration of intravenous thrombolysis.

TIA transient ischaemic attack; MI myocardial infarction; CHF congestive heart failure; NIHSS National Institute of Health Stroke Scale; IVT intravenous thrombolysis.

|  | **Good functional outcome**  **[n. 214]** | **Death or disability**  **[n. 277]** | ***p value*** |
| --- | --- | --- | --- |
| Age | 72.5±11.6 | 73.9±11.4 | 0.2 |
| Sex (M) | 121 (56.5%) | 149 (53.8%) | 0.6 |
| Previous stroke or TIA | 36 (16.8%) | 58 (20.9%) | 0.3 |
| Atrial fibrillation | 56 (26.2%) | 87 (31.4%) | 0.2 |
| Hypertension | 135 (63.1%) | 181 (65.3%) | 0.6 |
| Diabetes mellitus | 41 (19.2%) | 62 (22.4%) | 0.4 |
| Hyperlypidemia | 77 (36.0%) | 98 (35.4%) | 0.9 |
| Alcoholism | 19 (8.9%) | 26 (9.4%) | 0.9 |
| Current smoker | 44 (20.6%) | 44 (15.9%) | 0.2 |
| MI history | 32 (15.0%) | 36 (13.0%) | 0.4 |
| CHF history | 17 (7.9%) | 21 (7.6%) | 0.9 |
| NIHSS on admission | 5.8±3.7 | 10.5±6.6 | **<0.001** |
| Antiplatelet on admission | 63 (29.4%) | 86 (31.1%) | 0.7 |
| Anticoagulant on admission | 23 (10.7%) | 52 (18.8%) | **0.02** |
| Statins on admission | 55 (25.7%) | 67 (24.2%) | 0.7 |
| Linfonodal involvement | 110 (51.4%) | 164 (59.2%) | 0.2 |
| Metastasis | 82 (38.3%) | 152 (54.9%) | **<0.001** |
| IVT | 114 (53.3%) | 102 (36.8%) | **<0.001** |

**Table S3.** Univariate analysis on primary outcomes at 90 days.

TIA transient ischaemic attack; MI myocardial infarction; CHF congestive heart failure; NIHSS National Institute of Health Stroke Scale; IVT intravenous thrombolysis

|  | **O.R. [95% CI]** | ***P*** |
| --- | --- | --- |
| Age | 0.99 [0.96 - 1.01] | 0.27 |
| Sex (M) | 0.93 [0.48 – 1.80] | 0.82 |
| Previous stroke/TIA | 1.79 [0.85 – 3.77] | 0.13 |
| Atrial fibrillation | 1.43 [0.57 – 3.60] | 0.45 |
| Hypertension | 0.92 [0.50 – 1.71] | 0.80 |
| Diabetes mellitus | 0.78 [0.40 – 1.56] | 0.48 |
| Hyperlipidemia | 1.01 [0.52 – 1.99] | 0.97 |
| Alcoholism | 0.78 [0.32 – 1.90] | 0.58 |
| Current smoker | 1.11 [0.56 – 2.21] | 0.77 |
| MI history | 1.71 [0.71 – 4.15] | 0.23 |
| CHF history | 1.71 [0.54 – 5.41] | 0.36 |
| NIHSS on admission | 0.81 [0.76 – 0.86] | **<0.001** |
| Antiplatelets on admission | 0.61 [0.29 – 1.29] | 0.19 |
| Anticoagulants on admission | 0.27 [0.10 – 0.69] | **0.006** |
| Statins on admission | 1.06 [0.48 – 2.35] | 0.89 |
| Linfonodal involvement | 0.76 [0.40 – 1.44] | 0.39 |
| Metastasis | 0.71 [0.39 – 1.30] | 0.27 |
| IVT | 2.56 [1.45 – 4.52] | **0.001** |

**Table S4.** Multivariable analysis on good functional outcome at 90 days.

TIA transient ischaemic attack; MI myocardial infarction; CHF congestive heart failure; NIHSS National Institute of Health Stroke Scale; IVT intravenous thrombolysis

|  | **O.R. [95% CI]** | ***P*** |
| --- | --- | --- |
| Age | 1.00 [0.98 - 1.03] | 0.78 |
| Sex (M) | 0.95 [0.50 – 1.82] | 0.88 |
| Previous stroke/TIA | 0.52 [0.25 – 1.09] | 0.08 |
| Atrial fibrillation | 0.85 [0.34 – 2.10] | 0.72 |
| Hypertension | 1.06 [0.56 – 2.03] | 0.85 |
| Diabetes mellitus | 1.22 [0.60 – 2.47] | 0.59 |
| Hyperlipidemia | 1.73 [0.85 – 3.55] | 0.13 |
| Alcoholism | 0.86 [0.32 – 2.30] | 0.77 |
| Current smoker | 0.81 [0.38 – 1.71] | 0.57 |
| MI history | 1.21 [0.50 – 2.94] | 0.67 |
| CHF history | 0.21 [0.06 – 0.74] | **0.02** |
| NIHSS on admission | 1.09 [1.05 – 1.15] | **<0.001** |
| Antiplatelets on admission | 1.12 [0.53 – 2.34] | 0.77 |
| Anticoagulants on admission | 1.18 [0.50 – 2.76] | 0.71 |
| Statins on admission | 0.78 [0.34 – 1.78] | 0.55 |
| Linfonodal involvement | 1.33 [0.67 – 2.66] | 0.42 |
| Metastasis | 1.86 [0.97 – 3.57] | 0.06 |
| IVT | 0.41 [0.22 – 0.74] | **0.003** |

**Table S5.** Multivariable analysis on mortality at 90 days.

TIA transient ischaemic attack; MI myocardial infarction; CHF congestive heart failure; NIHSS National Institute of Health Stroke Scale; IVT intravenous thrombolysis

|  | **O.R. [95% CI]** | ***P*** |
| --- | --- | --- |
| Age | 0.99 [0.96 - 1.02] | 0.51 |
| Sex (M) | 0.45 [0.23 – 0.89] | **0.02** |
| Previous stroke/TIA | 2.34 [1.06 – 5.18] | **0.04** |
| Atrial fibrillation | 0.96 [0.44 – 2.12] | 0.92 |
| Hypertension | 1.13 [0.55 – 2.31] | 0.74 |
| Diabetes mellitus | 0.70 [0.30 – 1.64] | 0.41 |
| Hyperlipidemia | 0.50 [0.20 – 1.24] | 0.13 |
| Alcoholism | 0.75 [0.20 – 2.83] | 0.67 |
| Current smoker | 0.37 [0.13 – 1.06] | 0.07 |
| MI | 0.71 [0.23 – 2.12] | 0.54 |
| CHF | 1.48 [0.46 – 4.80] | 0.51 |
| NIHSS on admission | 1.07 [1.01 – 1.12] | **0.02** |
| Antiplatelets on admission | 1.51 [0.64 – 3.56] | 0.34 |
| Anticoagulants on admission | 3.42 [1.31 – 8.93] | **0.01** |
| Statins on admission | 1.05 [0.39 – 2.85] | 0.92 |
| Linfonodal involvement | 1.36 [0.63 – 2.94] | 0.44 |
| Metastasis | 1.03 [0.51 – 2.10] | 0.93 |
| IVT | 4.13 [1.99 – 8.57] | **<0.001** |

**Table S6.** Multivariable analysis on any haemorrhagic event at 90 days.

TIA transient ischaemic attack; MI myocardial infarction; CHF congestive heart failure; NIHSS National Institute of Health Stroke Scale; IVT intravenous thrombolysis

|  | **O.R. [95% CI]** | ***p value*** |
| --- | --- | --- |
| Age | 0.99 [0.96 - 1.02] | 0.47 |
| Sex (M) | 0.54 [0.26 – 1.14] | 0.11 |
| Previous stroke/TIA | 2.24 [0.94 – 5.31] | 0.07 |
| Atrial fibrillation | 0.74 [0.30 – 1.81] | 0.51 |
| Hypertension | 0.89 [0.41 – 1.95] | 0.77 |
| Diabetes mellitus | 0.77 [0.30 – 1.99] | 0.60 |
| Hyperlipidemia | 0.68 [0.25 – 1.84] | 0.44 |
| Alcoholism | 0.52 [0.10 – 2.62] | 0.43 |
| Current smoker | 0.53 [0.18 – 1.55] | 0.25 |
| MI | 0.50 [0.14 – 1.82] | 0.29 |
| CHF | 2.63 [0.79 – 8.76] | 0.12 |
| NIHSS on admission | 1.06 [1.00 – 1.13] | 0.37 |
| Antiplatelets on admission | 1.90 [0.74 – 4.86] | 0.18 |
| Anticoagulants on admission | 4.51 [1.55 – 13.16] | **0.006** |
| Statins on admission | 0.81 [0.27 – 2.41] | 0.70 |
| Linfonodal involvement | 1.64 [0.67 – 4.03] | 0.28 |
| Metastasis | 1.51 [0.68 – 3.36] | 0.31 |
| IVT | 4.85 [2.09 – 11.22] | **<0.001** |

**Table S7.** Multivariable analysis on intracranial haemorrhage at 90 days.

TIA transient ischaemic attack; MI myocardial infarction; CHF congestive heart failure; NIHSS National Institute of Health Stroke Scale; IVT intravenous thrombolysis.

|  | **Lung**  **[tot. 118]** | **Genitourinary [tot. 96]** | **Breast**  **[tot. 69]** | **Colorectal [tot. 54]** | **Pancreatic/**  **hepatobiliary**  **[tot. 49]** | **Upper**  **gastrointestinal**  **[tot. 42]** |
| --- | --- | --- | --- | --- | --- | --- |
| **Good functional outcome** | 46 (39.0%) | 37 (38.5%) | 38 (55.1%) | 21 (38.9%) | 11 (22.4%) | 13 (31.0%) |
| **Mortality** | 38 (32.2%) | 17 (17.7%) | 8 (11.6%) | 12 (22.2%) | 23 (46.9%) | 12 (28.6%) |
| **Any haemorrhagic event** | 17 (14.4%) | 15 (15.6%) | 4 (5.8%) | 8 (14.8%) | 4 (8.2%) | 8 (19.0%) |
| **Intracranial haemorrhage** | 16 (13.6%) | 10 (10.4%) | 4 (5.8%) | 7 (13.0%) | 3 (6.1%) | 2 (4.8%) |

**Table S8.** Frequencies of primary and secondary outcomes in different primary sites of active cancer.

**
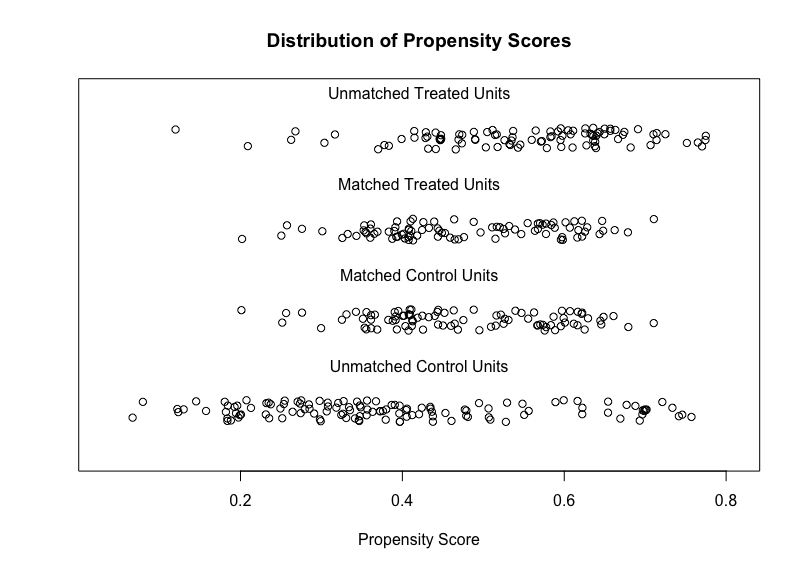
**

**Figure 1.** Distribution of propensity scores in patients treated and not treated with intravenous thrombolysis.

**
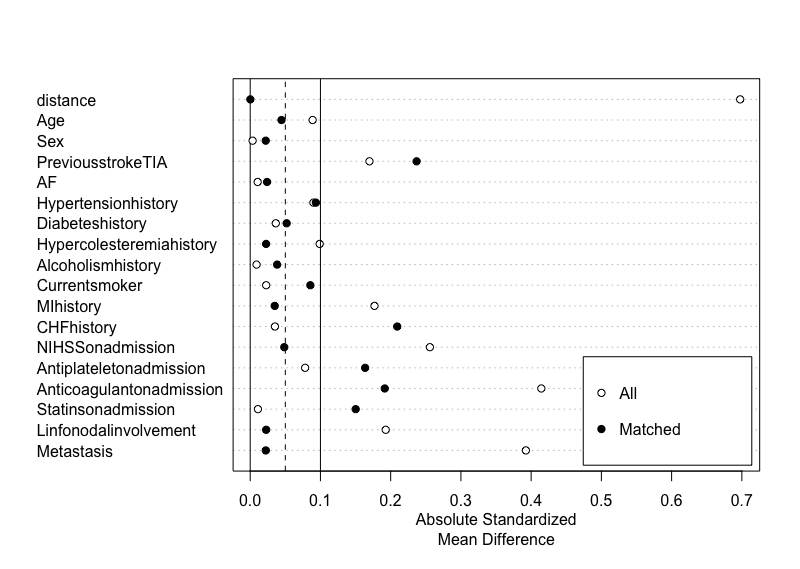
**

**Figure 2.** Love plot representing the balance of the variables included in the propensity score matching analysis.

|  | **IVT (n. 91)** | **No IVT (n. 91)** | **SMD** | **Total (n. 182)** | ***p value*** |
| --- | --- | --- | --- | --- | --- |
| Age | 73.58 (9.92) | 73.08 (10.35) | 0.0446 | 73.33 (10.11) | 0.89 |
| Sex (M) | 41 (45.1%) | 42 (46.2%) | -0.0221 | 83 (45.6%) | 0.88 |
| Previous stroke/TIA | 14 (15.4%) | 22 (24.2%) | -0.2369 | 36 (19.8%) | 0.88 |
| Atrial fibrillation | 22 (24.2%) | 23 (25.3%) | -0.0240 | 45 (24.7%) | 0.86 |
| Hypertension | 62 (68.1%) | 58 (63.7%) | 0.0935 | 120 (65.9%) | 0.53 |
| Diabetes mellitus | 21 (23.1%) | 19 (20.9%) | 0.0519 | 40 (22.0%) | 0.72 |
| Hyperlipidemia | 33 (36.3%) | 32 (35.2%) | 0.0226 | 65 (35.7%) | 0.88 |
| Alcoholism | 8 (8.8%) | 9 (9.9%) | -0.0383 | 17 (9.3%) | 0.80 |
| Current smoker | 18 (19.8%) | 15 (16.5%) | 0.0857 | 33 (18.1%) | 0.56 |
| MI | 11 (12.1%) | 12 (13.2%) | -0.0349 | 23 (12.6%) | 0.82 |
| CHF | 5 (5.5%) | 10 (11.0%) | -0.2093 | 15 (8.2%) | 0.18 |
| NIHSS on admission | 8.34 (4.79) | 8.09 (6.86) | 0.0485 | 8.21 (5.90) | 0.10 |
| Antiplatelets on admission | 24 (26.4%) | 31 (34.1%) | -0.1636 | 55 (30.2%) | 0.26 |
| Anticoagulants on admission | 10 (11.0%) | 5 (5.5%) | 0.1916 | 15 (8.2%) | 0.18 |
| Statins on admission | 22 (24.2%) | 28 (30.8%) | -0.1502 | 50 (27.5%) | 0.32 |
| Linfonodal involvement | 65 (71.4%) | 64 (70.3%) | 0.0227 | 129 (70.9%) | 0.87 |
| Metastasis | 48 (52.7%) | 49 (53.8%) | -0.0223 | 97 (53.3%) | 0.88 |

**Table S9.** Characteristics of the patients after propensity score matching.

IVT intravenous thrombolysis; SMD standardized mean difference; TIA transient ischaemic attack; MI myocardial infarction; CHF congestive heart failure; NIHSS National Institute of Health Stroke Scale;
